# The exploration of perioperative hypotension subtypes: a prospective, single cohort, observational pilot study

**DOI:** 10.3389/fmed.2024.1358067

**Published:** 2024-06-17

**Authors:** Xu Zhao, Yuanjia Zhang, Mengjia Kou, Zhongxing Wang, Qiulan He, Zhishuang Wen, Jingyuan Chen, Yiyan Song, Shihui Wu, Chanyan Huang, Wenqi Huang

**Affiliations:** Department of Anesthesiology, The First Affiliated Hospital, Sun Yat-sen University, Guangzhou, China

**Keywords:** hypotension, cardiac output, perioperative, hemodynamic, myocardial injury after noncardiac surgery, acute kidney injury, pilot study

## Abstract

**Background:**

Hypotension is a risk factor for postoperative complications, but evidence from randomized trials does not support that a higher blood pressure target always leads to optimized outcomes. The heterogeneity of underlying hemodynamics during hypotension may contribute to these contradictory results. Exploring the subtypes of hypotension can enable optimal management of intraoperative hypotension.

**Methods:**

This is a prospective, observational pilot study. Patients who were ≥ 45 years old and scheduled to undergo moderate-to-high-risk noncardiac surgery were enrolled in this study. The primary objective of this pilot study was to investigate the frequency and distribution of perioperative hypotension and its subtypes (hypotension with or without cardiac output reduction). The exposure of hypotension and its subtypes in patients with and without myocardial or acute kidney injury were also explored.

**Results:**

Sixty patients were included in the analysis. 83% (50/60) of the patients experienced perioperative hypotension. The median duration of hypotension for each patient was 8.0 [interquartile range, 3.1–23.3] minutes. Reduced cardiac output was present during 77% of the hypotension duration. Patients suffering from postoperative myocardial or acute kidney injury displayed longer duration and more extensive exposure in all hypotension subtypes. However, the percentage of different hypotension subtypes did not differ in patients with or without postoperative myocardial or acute kidney injury.

**Conclusion:**

Perioperative hypotension was frequently accompanied by cardiac output reduction in moderate-to-high-risk noncardiac surgical patients. However, due to the pilot nature of this study, the relationship between hypotension subtypes and postoperative myocardial or acute kidney injury still needs further exploration.

**Clinical trial registration:**

https://www.chictr.org.cn/showprojEN.html?proj=134260, CTR2200055929.

## Introduction

Blood pressure management is an everlasting topic in perioperative medicine. Varying degrees of hypotension are commonly encountered during anesthesia and surgery (1). In recent years, studies have revealed the association between perioperative hypotension and organ injury (2). Hypotension can decrease perfusion pressure and affect organ perfusion, eventually leading to organ or tissue ischemia (3). Studies based on large-sample cohorts have shown that perioperative hypotension increases the incidence of postoperative myocardial injury (4–7), kidney injury (5, 8–10), stroke (11, 12), and also the mortality rate (3, 4, 6). On the contrary, there are studies that suggested no association between hypotension and adverse outcomes (13, 14); whereas results from randomized controlled trials did not consistently prove that maintaining higher blood pressure improves postoperative outcomes (15).

These contradictory findings can be attributed to many reasons, among which the heterogeneity of hypotension itself may be a contributing factor (16). A recent study using a machine learning algorithm suggested that there are distinct endotypes of intraoperative hypotension (17). Depending on the relative changes between the perfusion pressure and the regional vascular resistance, hypotension may not affect or even increase organ perfusion. Therefore, exploring the underlying hemodynamics during hypotension may provide new insights into perioperative blood pressure management.

Cardiac output and systemic vascular resistance are the leading determinants of blood pressure (18). Previous reviews also pointed out that cardiac output is associated with organ perfusion (16, 19, 20), and the use of a cardiac output-guided hemodynamic therapy might be associated with a reduction in postoperative complication rates (21). Therefore, we focused on the relationship between cardiac output and blood pressure in this study. Hypotension can be classified into two subtypes depending on whether the cardiac output is decreased. Currently, studies investigating the frequency and distribution of hypotension with or without cardiac output reduction are lacking. In addition, as adequate organ perfusion is crucial to prevent organ ischemia, exploring the association between hypotension subtypes and postoperative ischemic injury is warranted.

### Objectives

The primary objective of this study was to investigate the frequency and distribution of hypotension and its subtypes (hypotension with or without cardiac output reduction) in patients who underwent moderate-to-high-risk noncardiac surgery. The secondary objectives were to compare the burden [measured by the total duration and area under the curve (AUC)] of hypotension and its subtypes in patients with or without myocardial or acute kidney injury, and explore whether patients with myocardial or acute kidney injury had experienced a higher percentage of hypotension with reduced cardiac output.

## Methods

This is a prospective, single cohort, observational pilot study. This study was approved by the Institutional Review Board of the First Affiliated Hospital of Sun Yat-sen University (Chairperson: Churong Yan) before the recruitment of any subject (No. [2021]-764; approval date: November 2, 2021). Written informed consent was obtained from all subjects participating in the trial. The trial was registered prior to patient enrollment in the Chinese Clinical Trial Registry [ChiCTR2200055929, Principal investigator: Xu Zhao, https://www.chictr.org.cn/showprojEN.html?proj=134260, Date of registration: November 16, 2021 (submission date); January 26, 2022 (publication date)]. The original study protocol is presented in the Supplementary files. This study has been reported in line with the Strengthening the Reporting of Observational Results in Epidemiology (STROBE) guidelines. Patients or the public were not involved in the design, conduct, reporting, or dissemination plans of our research.

### Participants

The inclusion criteria for the participants are: (1) age ≥ 45 years; (2) undergoes moderate- or high-risk noncardiac surgery (surgical risk classification based on the guidelines on noncardiac surgery (22), encompassing procedures such as gastrointestinal surgery, hepatobiliary surgery, urology surgery, esophagectomy, pneumonectomy, lung or liver transplantation); (3) undergoes general anesthesia; (4) surgery duration expected to last ≥2 h from skin incision; (5) planned postoperative hospitalization of ≥3 days; and (6) obtain written informed consent from patients.

The exclusion criteria are: (1) participates in another interventional study; (2) undergoes low-risk surgery only (e.g., superficial surgery, breast surgery, thyroid surgery, minor plastic surgery, transurethral resection of the prostate); (3) pregnant or nursing mothers; (4) undergoes emergency surgery; (5) suffers from severe cardiovascular comorbidities (clinically important intra-cardiac shunts, aortic stenosis with valve area ≤ 1.5 cm^2^, moderate to severe aortic regurgitation, moderate to severe mitral regurgitation, moderate to severe mitral stenosis, persistent atrial fibrillation, acute congestive heart failure); (6) previously received heart valve surgery, coronary artery bypass grafting, percutaneous coronary intervention, pacemaker implantation, or implantable cardioverter defibrillator implantation; (7) patient whom intraoperative mean arterial pressure target will be <65 mmHg (including controlled hypotension); (8) has an intra-aortic balloon pump or ventricular assist device; and (9) requires multiple vasoactive agents and a known diagnosis of ongoing active sepsis.

The patient’s medical records were reviewed to assess eligibility. Once the potential eligibility had been determined, the study was discussed with the patient. The patients who agreed to participate in this study then signed the informed consent form before their respective surgeries, and a sequential research ID was subsequently provided.

### Perioperative monitoring and anesthetic management

Routing monitoring consisted of electrocardiography, pulse oximetry, invasive/noninvasive blood pressure, end-tidal carbon dioxide, body temperature, and an electroencephalogram monitor (Narcotrend®; MonitorTechnik, Bad Bramstedt, Germany). The attending anesthesiologist in charge determined whether a patient required an arterial line or central venous catheter insertion. In addition, patients received a hemodynamic monitor (CNAP^®^; CNSystems Medizintechnik GmbH, Graz, Austria) to continuously obtain information for patients’ blood pressure, pulse rate, cardiac output, systemic vascular resistance, and stroke volume. The finger cuff of the CNAP® monitor was placed on the hand, while the cuff used for noninvasive blood pressure measurement was placed on the same upper arm for calibration (Supplementary Figure S1). The upper arm intermittent noninvasive blood pressure was used to calibrate the continuous finger blood pressure value automatically, and the calibration interval was set to 20 min. If an arterial line was placed in the radial artery, the finger cuff was applied at the contralateral side. The information on stroke volume, cardiac output, and systemic vascular resistance measurements was blinded for anesthesiologists.

The anesthetic regime was left at the discretion of the anesthesiologist in charge. Anesthesia was induced with propofol and remifentanil. Rocuronium or cisatracurium was administered, and tracheal intubation was performed. Anesthesia was maintained with either intravenous (propofol and remifentanil), combined intravenous-inhalational anesthetics (propofol, remifentanil, and sevoflurane), or combined epidural–general anesthetics (propofol, remifentanil, and ropivacaine administered through the epidural catheter). Propofol and sevoflurane were titrated to keep 95% spectral edge frequency within 8–12 Hz as guided by the electroencephalogram. Patient-controlled intravenous analgesia or patient-controlled epidural analgesia was provided after surgery.

The intervention for hypotension was initiated when patients’ mean arterial pressure stayed less than 65 mmHg for more than 1 min. However, for safety concerns, the intervention of hypotension was also initiated for patients with preoperative hypertension when their systemic blood pressure decreased to below 80% of baseline measures. Intraoperative hypotension was treated with a bolus of metaraminol or ephedrine, a continuous infusion of norepinephrine or dopamine, or a fluid bolus as clinically indicated.

### Data sources

A research laptop was used to capture all real-time monitoring data. Data recording started before anesthesia induction and stopped after extubation. Venous blood samples were drawn from each patient at 4 time points: (1) 24 h after surgery, (2) 48 h after surgery, (3) 72 h after surgery, and (4) 7 days after surgery or the last day before hospital discharge (if the length of postoperative stay was less than 7). The concentration of serum troponin T, troponin I, and creatinine were determined by researchers who were not involved in patient care.

### Variables

**Figure 1:**
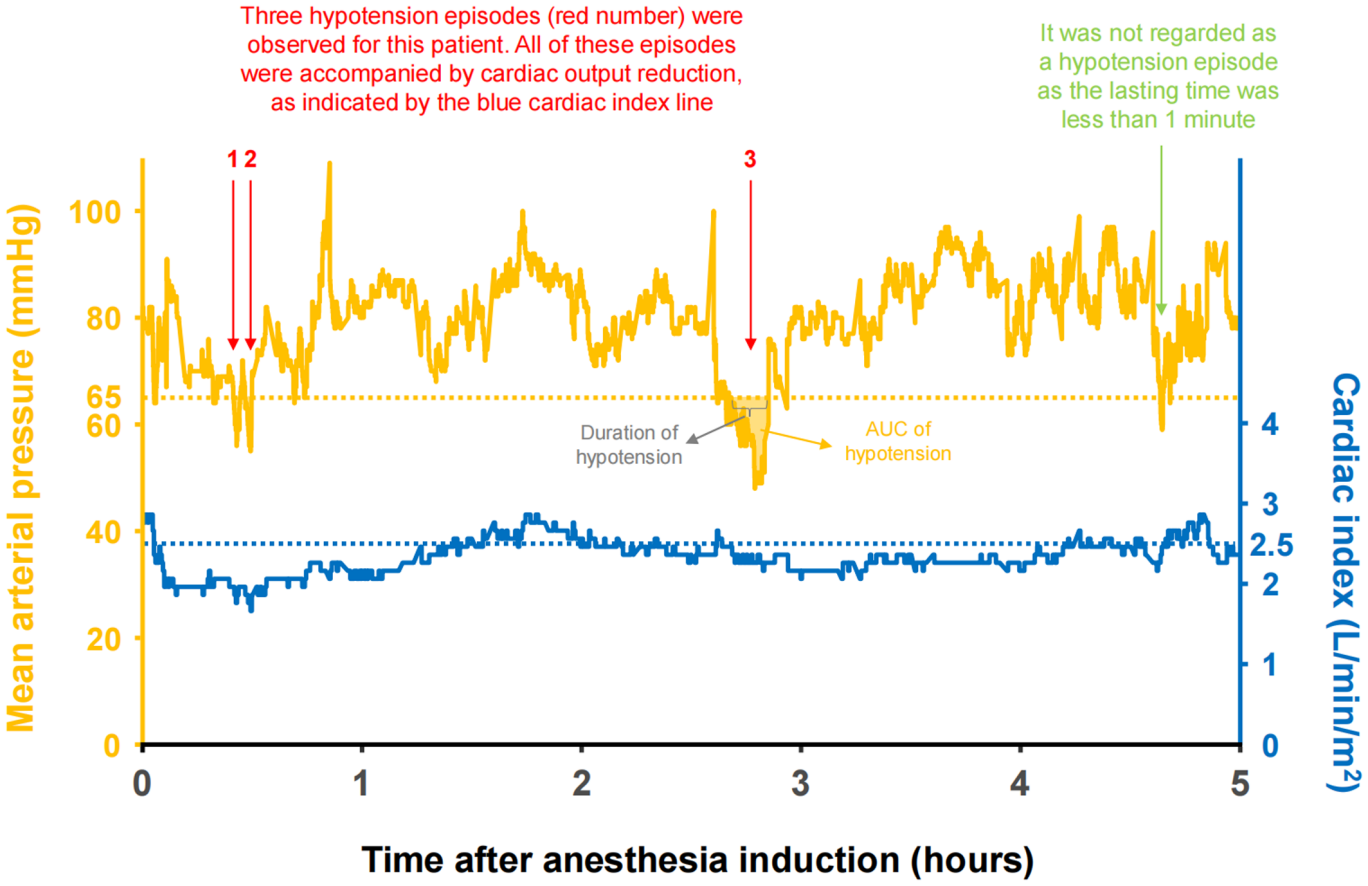
The actual tracing of mean arterial pressure (fluctuating orange line), threshold (straight lines) and area under the curve (shaded area) in one patient.

An episode of hypotension is defined by a mean arterial pressure of <65 mmHg for more than 1 min (Figure 1). Previous studies suggested that a mean arterial pressure of <65 mmHg is a threshold at which the incidence of postoperative myocardial injury and acute kidney injury increases (5). Our definition of hypotension is consistent with recent studies (23, 24). Every hypotension episode could be classified into different subtypes based on whether the hypotension episode was accompanied by cardiac output reduction (Figure 1). The hypotension was regarded as accompanied by a reduced cardiac output if the cardiac index is <2.5 L/min/m^2^ or < 10% below baseline when hypotension happens (25). In this study, we used the following variables to measure the burden of hypotension for each patient: (1) duration: the cumulative time for all hypotension episodes; (2) maximum duration of a single episode of hypotension; and (3) AUC: the area under the curve below the mean arterial pressure threshold (65 mmHg), reflecting the products of hypotension duration and extent. For example, if a patient experienced a mean arterial pressure of 55 mmHg for 10 min, the AUC of hypotension was (65–55 mmHg) * 10 min = 100 mmHg*min (26). A detailed explanation for these variables was presented in Figure 1, with an actual tracing of mean arterial pressure and cardiac index in one patient.

Postoperative myocardial injury is defined by a peak serum troponin T concentration of ≥0.03 ng·ml^−1^ without a nonischemic etiology to cause troponin T elevation, which is consistent with the guideline from the European Perioperative Clinical Outcome (EPCO) (27). Based on recommendations from the EPCO guideline, the concentration of troponin T was monitored daily within the first 3 days after surgery to accurately evaluate postoperative myocardial injury (27).

Postoperative acute kidney injury is defined according to the Kidney Disease: Improving Global Outcomes (KDIGO) criteria (28). Briefly, an acute kidney injury is defined as any of the following: (1) increase in serum creatinine to ≥0.3 mg/dL within 48 h; (2) increase in serum creatinine to ≥1.5 times of the baseline within 7 days after surgery; or (3) urine volume of <0.5 mL·kg^−1^·hour^−1^ for 6 h. The severity of acute kidney injury is also categorized as per the KDIGO criteria (28).

Missing data were dealt with depending on the types of data. For patient demographics, preoperative baseline, surgery-related information, intraoperative management, and medication, missing data were not imputed. If outcome data were missing at more than 1 time point, the patient was excluded from data analysis. When judging whether the patient with one missing outcome data had myocardial or kidney injuries, the patient was judged as having no injury if the troponin and creatine at other time points showed negative results and patients did not show specific symptoms. If data were missing for the continuous recording (blood pressure, heart rate, cardiac output, etc.) due to machine malfunction or unexpected stops, the patient was excluded from analysis. If only a few values (<10% of the recording time) were missing, they were imputed using the value before them. However, the vital signs were not imputed for more than 5 consecutive minutes.

### Bias

Efforts have been made to reduce bias during data collection. A training was conducted for all research staff before data acquisition to ensure consistency. Two researchers carried out the data collection process independently to ensure data accuracy. A quality control project (dealing with missing and abnormal data) was performed prior to data analysis.

### Study size

This was the first study to explore the frequency and distribution of different hypotension subtypes in the perioperative setting, and no previous data were available for a sample size calculation. Since this was a pilot study and traditional power calculation may not be appropriate (29), no formal sample size calculation was performed. Given the current surgical volume at our hospital, 60 participants were planned to be enrolled in this pilot study.

### Quantitative variables

All quantitative variables were prospectively collected using a predesigned case report form and subsequently transferred to an electronic data sheet by two researchers. The data sheet was de-identified before data analysis.

### Statistical methods

Continuous variables with a normal distribution are presented as mean and standard deviation; otherwise, they are presented as median and interquartile range. The normality of distribution was assessed using histograms and Q–Q plots. The categorical variables are presented as frequency and percentage.

The following analyses were done. (1) The frequency, duration and time distribution of hypotension and its subtypes during noncardiac surgery were described. (2) The burden of hypotension and its subtypes (duration and AUC of hypotension; explained in the Variables section) were compared in patients with or without postoperative myocardial injury and/or acute kidney injury. We calculated the percentages of different hypotension subtypes for each patient (the duration of different hypotension subtypes/the duration of total hypotension*100%), which could be used to measure the contribution of cardiac output reduction to hypotension.

Subgroup analyses were performed based on different patient populations. In addition, two sensitivity analyses were performed based on different hypotension thresholds (mean arterial pressure < 60 mmHg and 55 mmHg) to test the robustness of the primary analysis. All analyses were performed using the R software (version 3.6.0, R Foundation for Statistical Computing, Austria).

## Results

### Participants and perioperative information

**Figure 2:**
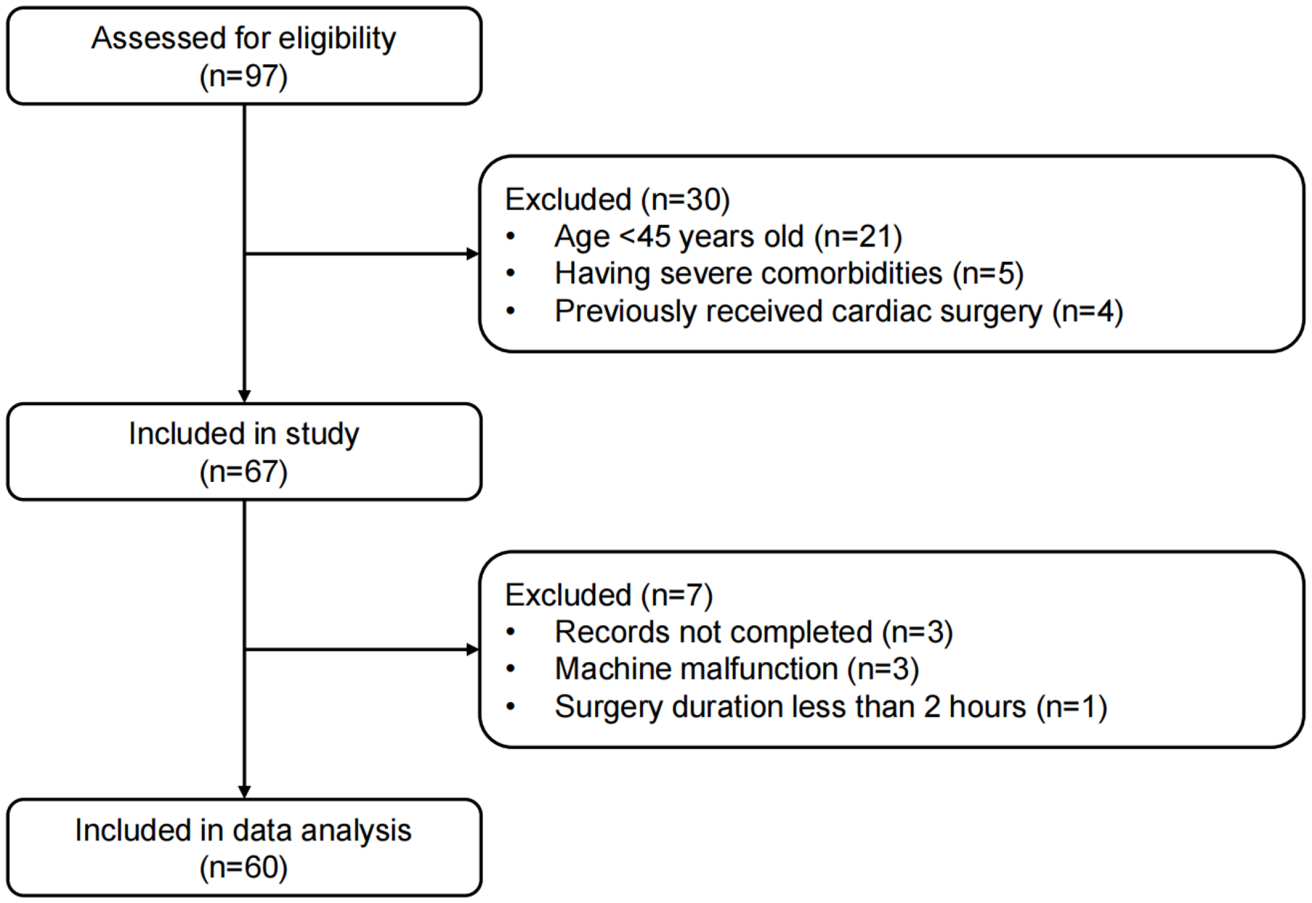
Flow diagram of patient enrollment and data analysis.

Between November 22, 2021 and March 7, 2022, 97 patients who were ≥ 45 years old and scheduled to undergo noncardiac surgery at our institute were screened, among which 67 patients were successfully enrolled in this study. Nevertheless, 7 patients were excluded from the final data analysis because of the following reasons: incomplete data records (*n* = 3), machine malfunction (*n* = 3), and surgery duration of less than 2 h (*n* = 1). Therefore, 60 patients were included in the final data analysis (Figure 2).

The characteristics and perioperative management information of the patients are listed in Table 1. The included subjects had a mean age of 63 years old at the time of surgery (standard deviation, 10 years), and 65.0% were male. Most patients received abdominal surgeries, with hepatectomy (36.7%), colorectal resection (18.3%) and gastrectomy (16.7%) being the most common types of surgery. All patients received general anesthesia, among which 48.3% received combined epidural–general anesthesia. Most patients received norepinephrine (88.3%) or metaraminol (76.7%) during surgery; whereas only 25% of the patients received dopamine, and 13.3% received ephedrine treatments.

**Table 1:** Perioperative data.^a^

Variable	Total (*n* = 60)	With postoperative MI or AKI (*n* = 14)	Without postoperative MI or AKI (*n* = 46)
Demographics			
Age, year	62.7 ± 9.6	61.0 ± 11.9	63.3 ± 8.9
Sex, male (%)	39 (65.0)	7 (50.0)	32 (69.6)
Body mass index, kg/m^2^	22.5 ± 2.9	22.3 ± 2.4	22.6 ± 3.0
Current smoker, no. (%)	18 (30.0)	4 (28.6)	14 (30.4)
ASA Physical Status, no. (%)			
II	3 (5.0)	1 (7.1)	2 (4.3)
III	57 (95.0)	13 (92.9)	44 (95.7)
Comorbidities, no. (%)			
Hypertension	16 (26.7)	4 (28.6)	12 (26.1)
Class I	9 (15.0)	2 (50.0)	7 (58.3)
Class II	4 (6.7)	0 (0.0)	4 (33.3)
Class III	3 (5.0)	2 (50.0)	1 (8.3)
Coronary heart disease	1 (1.7)	0 (0.0)	1 (2.2)
COPD	1 (1.7)	0 (0.0)	1 (2.2)
Diabetes	10 (16.7)	2 (14.3)	8 (17.4)
Gastroduodenal ulcer	3 (5.0)	0 (0.0)	3 (6.5)
Viral hepatitis	14 (23.3)	4 (28.6)	10 (21.7)
Chronic kidney disease	0 (0.0)	0 (0.0)	0 (0.0)
History of medication, no. (%)			
ACEI or ARB	3 (5.0)	2 (14.3)	1 (2.2)
Beta blocker	1 (1.7)	0 (0.0)	1 (2.2)
Calcium channel blocker	11 (18.3)	3 (21.4)	8 (17.4)
Diuretic	2 (3.3)	2 (14.3)	0 (0.0)
Statins	2 (3.3)	1 (7.1)	1 (2.2)
Type of surgery, no. (%)			
Hepatectomy	22 (36.7)	7 (50.0)	15 (32.6)
Colorectal resection	11 (18.3)	2 (14.3)	9 (19.6)
Gastrectomy	10 (16.7)	2 (14.3)	8 (17.4)
Esophagectomy	7 (11.7)	1 (7.1)	6 (13.0)
Pancreaticoduodenectomy	5 (8.3)	2 (14.3)	3 (6.5)
Lobectomy	4 (6.7)	0 (0.0)	4 (8.7)
Biliary tract surgery	1 (1.7)	0 (0.0)	1 (2.2)
Surgical technique, no. (%)			
Open	33 (55.0)	8 (57.1)	25 (54.3)
Laparoscopic-assisted	26 (43.3)	5 (35.7)	21 (45.7)
Robotic-assisted	1 (1.7)	1 (7.1)	0 (0.0)
Preoperative laboratory results			
Hemoglobin, g/L	123 ± 23	122 ± 23	123 ± 23
Creatine, umol/L	74 [61–87]	63 [59–95]	75 [63–87]
Preoperative hemodynamic baseline			
Systolic blood pressure, mmHg	129 ± 22	124 ± 15	130 ± 23
Diastolic blood pressure, mmHg	79 ± 12	78 ± 14	79 ± 12
Mean arterial pressure, mmHg	98 ± 16	96 ± 14	98 ± 16
Heart rate, beat/min	74 ± 11	71 ± 8	74 ± 12
Cardiac index, L/min/m^2^	2.8 [2.4–3.1]	2.8 [2.4–3.0]	2.8 [2.5–3.1]
SVRI, dynes*sec/cm^5^/m^2^	2,561 ± 544	2,499 ± 646	2,579 ± 516
Stroke volume, ml	63 [56–73]	65 [63–75]	61 [55–72]
Anesthesia managements			
Duration of anesthesia, min	310 [245–368]	326 [278–454]	320 [252–369]
Duration of surgery, min	264 [215–298]	266 [218–381]	264 [214–295]
Epidural analgesia, no. (%)	29 (48.3)	7 (50.0)	22 (47.8)
Arterial line insertion, no. (%)	47 (78.3)	13 (92.9)	34 (73.9)
Central venous catheter, no. (%)	54 (90.0)	14 (100.0)	40 (87.0)
Medication administered during surgery			
Propofol, mg	1,135 ± 370	1,064 ± 404	1,156 ± 361
Sevoflurane, no. (%)	25 (41.7)	7 (50.0)	18 (39.1)
Remifentanil, mg	2.5 ± 0.8	2.6 ± 0.8	± 0.7
Sufentanil, ug	29 ± 15	25 ± 14	31 ± 15
Cisatracurium, no. (%)	10 (16.7)	1 (7.1)	9 (19.6)
Rocuronium, no. (%)	50 (83.3)	13 (92.9)	37 (80.4)
Dexmedetomidine, no. (%)	49 (81.7)	9 (64.3)	40 (87.0)
Flurbiprofen axetil, no. (%)	29 (48.3)	8 (57.1)	21 (45.7)
Norepinephrine			
Number of patients used (%)	53 (88.3)	12 (85.7)	41 (89.1)
Median [IQR], ug	1,078 [557–1745]	1873 [629–3,146]	1,065 [557–1,673]
Metaraminol			
Number of patients used (%)	46 (76.7)	11 (78.6)	35 (76.1)
Median [IQR], mg	1.0 [0.6–1.5]	1.2 [0.6–1.8]	0.9 [0.6–1.5]
Dopamine			
Number of patients used (%)	15 (25.0)	4 (28.6)	11 (25.0)
Median [IQR], mg	47 [35–76]	84 [77–89]	42 [26–48]
Ephedrine			
Number of patients used (%)	8 (13.3)	3 (21.4)	5 (10.9)
Median [IQR], mg	13 [10–15]	15 [10–15]	10 [10–15]
Input and output during surgery			
Crystalloid, ml	2,250 [1800–3,200]	2,500 [2200–3,475]	2,200 [1800–3,100]
Colloid, ml	600 [500–1,000]	600 [500–1,000]	600 [500–1,000]
Transfusion, no. (%)	9 (15.0)	3 (21.4)	6 (13.0)
Estimated blood loss, ml	100 [50–200]	200 [100–575]	100 [50–200]
Urine output, ml	800 [400–1,400]	525 [400–1,515]	800 [438–1,400]
Outcome			
Postoperative myocardial injury or acute kidney injury, *n* (%)	14 (23.3)	14 (23.3)	–
Myocardial injury within postoperative 3-day, *n* (%)	8 (13.3)	8 (13.3)	–
Acute kidney injury within postoperative 7-day, *n* (%)	6 (10.0)	6 (10.0)	–
Unplanned intensive care unit admission, *n* (%)	3 (5.0)	1 (7.1)	2 (4.3)
Postoperative 30-day mortality, *n* (%)	0 (0.0)	0 (0.0)	0 (0.0)

MI, myocardial injury; AKI, acute kidney injury; ASA, American Society of Anesthesiologists; COPD, Chronic obstructive pulmonary disease; ACEI, angiotensin-converting enzyme inhibitors; ARB, angiotensin II receptor blockers; SVRI, systemic vascular resistance index; IQR, interquartile range. “–” indicates the value was not calculable.

a Data were presented as mean ± standard deviation, median [IQR], or frequency (percentage).

### The frequency and distribution of hypotension and its subtypes

**Figure 3:**
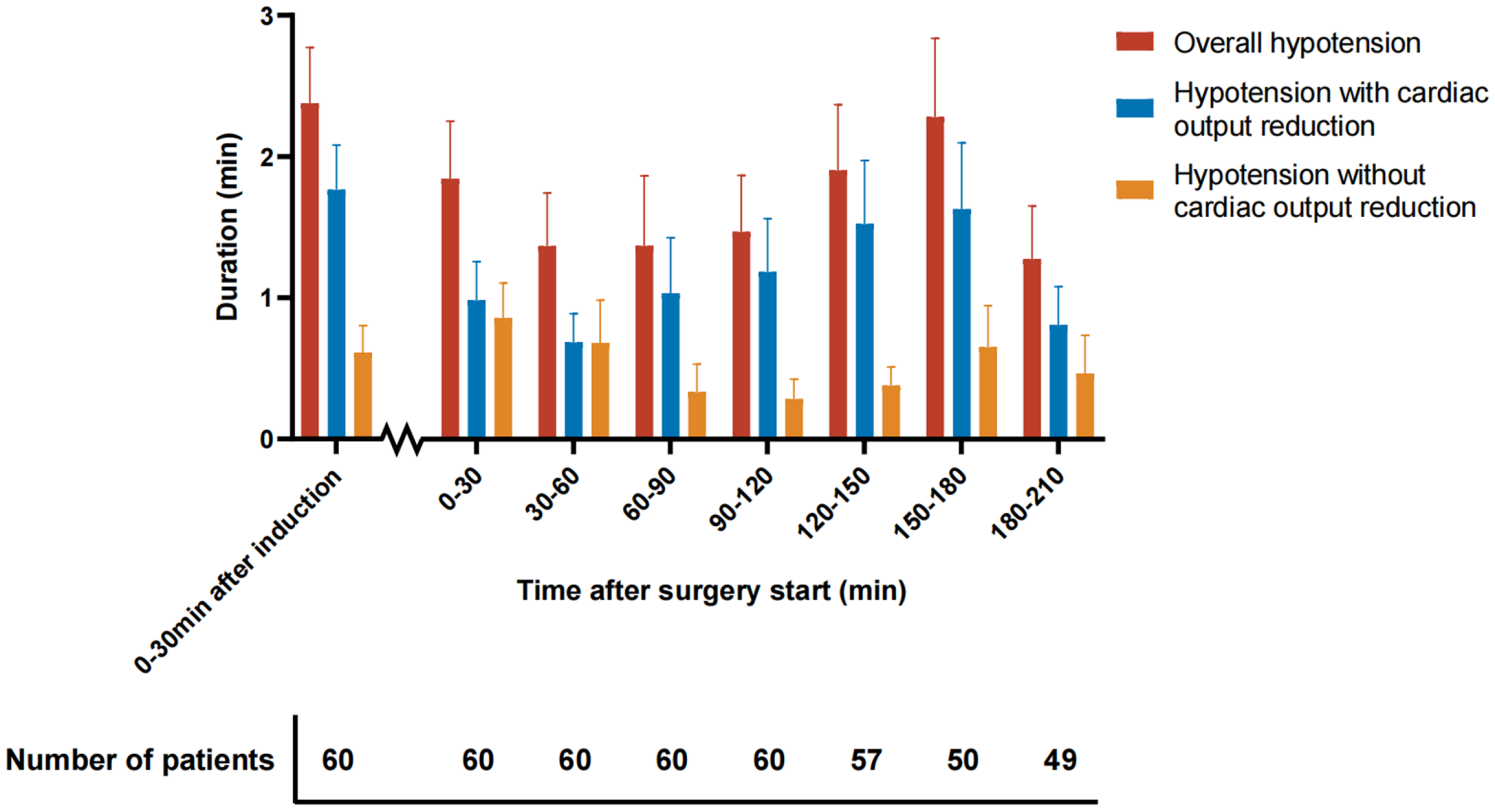
Time distribution of perioperative hypotension. Data are presented as mean ± standard error of the mean. The number below the x-axis indicates the number of patients analyzed at each time period.

The frequency and duration of hypotension and its subtypes are presented in Table 2. 50 (83.3%) out of the 60 patients who were included in this study experienced perioperative hypotension. The median frequency of hypotension (count of hypotension episodes) for each patient was 3 [interquartile range, 1–8], and the median duration of hypotension for each patient was 8.0 [interquartile range, 3.1–23.3] minutes. Regarding the underlying hemodynamic mechanisms, reduced cardiac output was present during 77% of the hypotension duration (Table 2). The median duration of hypotension with and without cardiac output reduction was 5.8 [interquartile range, 1.4–14.9] and 0.6 [interquartile range, 0.0–7.5] minutes, respectively. The hypotension yielded a higher frequency within 30 min after anesthesia induction, compared to hypotension occurrence during surgery. Cardiac output-reduced hypotension is the primary type of hypotension within 30 min after anesthesia induction (Figure 3).

**Table 2:** Frequency and duration of overall hypotension and hypotension subtypes.^a^

Variable	Total (*n* = 60)	With postoperative MI or AKI (*n* = 14)	Without postoperative MI or AKI (*n* = 46)	*p* value^b^
Overall hypotension				
Patients with hypotension, *n* (%)	50 (83.3)	11 (78.6)	39 (84.8)	0.59
Frequency of hypotension^c^, *n*	3 [1–8]	3 [1–9]	3 [1–8]	0.90
Duration of hypotension, min	8.0 [3.1–23.3]	13.6 [2.3–28.3]	7.4 [3.6–22.4]	0.82
Maximum duration of a single episode of hypotension, min	4.5 ± 3.5	5.1 ± 4.4	4.4 ± 3.3	0.53
AUC of hypotension^d^, mmHg*min	4,413 [759–11,028]	5,670 [789–11,494]	3,699 [922–10,147]	0.71
Hypotension subtypes				
Hypotension with reduced cardiac output^e^				
Duration, min	5.8 [1.4–14.9]	8.6 [0.4–14.6]	5.6 [1.8–14.3]	0.96
Maximum duration of a single episode, min	3.2 ± 2.9	3.6 ± 3.7	3.1 ± 2.7	0.60
AUC^f^, mmHg*min	3,158 [398–6,759]	4,051 [70–7,842]	2,999 [433–6,009]	0.99
Hypotension without reduced cardiac output^g^				
Duration, min	0.6 [0.0–7.5]	2.3 [0.0–11.4]	0.0 [0.0–6.7]	0.27
Maximum duration of a single episode, min	1.3 ± 2.4	1.5 ± 3.0	1.3 ± 2.2	0.78
AUC, mmHg*min	408 [0–4,038]	766 [0–5,818]	0 [0–3,394]	0.28
Percentage of hypotension subtypes^h^ (The duration of hypotension subtypes / duration of total hypotension * 100% for each patient)				
Percentage of hypotension with reduced cardiac output, %	77 [52–100]	77 [51–86]	76 [54–100]	0.66
Percentage of hypotension without reduced cardiac output, %	23 [0–48]	23 [14–49]	24 [0–46]	0.66

MI, myocardial injury; AKI, acute kidney injury; AUC, area under the curve; MAP, mean arterial pressure.

a Data were presented as frequency (percentage), mean ± standard deviation, or median [interquartile range].

b The *p* values were calculated using chi-square test, two-sample t test, or Mann–Whitney U test as appropriate.

c The frequency of hypotension indicated the total count of hypotension episodes for each patient.

d AUC indicates the area under the curve below the MAP threshold (65 mmHg), reflecting the products of hypotension duration and extent.

e Hypotension with reduced cardiac output indicates that hypotension episodes (MAP <65 mmHg lasting for at least 1 min) were accompanied by reduced cardiac output (cardiac index <2.5 L/min/m^2^ or < 10% below preoperative baseline). The MAP and cardiac index were simultaneously monitored and recorded.

f The AUC of hypotension with reduced cardiac output indicates the AUC below the MAP threshold (65 mmHg), while the low MAP was accompanied by a reduced cardiac output. It reflects the products of cardiac output reduction-related hypotension duration and extent.

g Hypotension without reduced cardiac output indicates the hypotension episodes were not accompanied by reduced cardiac output (cardiac index ≥2.5 L/min/m^2^ or ≥ 90% of the preoperative baseline).

h The percentage of hypotension with reduced cardiac output equals the duration of hypotension with reduced cardiac output / duration of total hypotension * 100% for each patient. However, we excluded the patients with a total hypotension duration <5 min when calculating the percentage, considering that short episodes might cause extreme percentage (0% or 100%) and biased the results.

Considering that the distribution of hypotension could vary among different patient populations, the frequency and duration of hypotension were further explored based on patients’ age, sex, body mass index, coexisting hypertension, coexisting diabetes, preoperative use of calcium channel blocker, type of surgery, surgical technique and epidural analgesia (Table 3). The results showed that most of the hypotension periods were accompanied by reduced cardiac output (median percentage of hypotension with reduced cardiac output ranges from 60 to 100%) regardless of the patient subgroup. Patients with older age (≥ 60 years vs. < 60 years, median 81% vs. 76%), having hypertension (yes vs. no, median 86% vs. 76%), having diabetes (yes vs. no, median 86% vs. 77%), using preoperative calcium channel blocker (yes vs. no, median 86% vs. 76%), and not receiving epidural analgesia (yes vs. no, median 63% vs. 78%) showed higher percentages of hypotension with reduced cardiac output. Patients who underwent esophagectomy (100%) and lobectomy (96%) had higher percentages of hypotension with reduced cardiac output than patients who underwent major abdominal surgeries (median percentages range from 60 to 76%).

**Table 3:** Subgroup analysis.^a^

	Incidence of postoperative MI or AKI (events/total number), %	Duration of anesthesia, min	Duration of hypotension, min	Duration of hypotension with reduced cardiac output^b^, min	Percentage of hypotension with reduced cardiac output^c^, %
Age					
≥ 60 years	8/34 (23.5)	328 [244–362]	10.0 [4.7–22.4]	7.1 [1.9–15.2]	81 [54–100]
< 60 years	6/26 (23.1)	297 [256–367]	5.7 [2.1–24.9]	3.3 [1.5–11.4]	76 [22–100]
*p* value for interaction^d^		0.98	0.18	0.24	0.91
Sex					
Female	7/21 (33.3)	331 [268–361]	11.2 [4.6–31.0]	7.3 [1.7.0–12.8]	77 [42–100]
Male	7/39 (17.9)	304 [238–370]	6.7 [2.2–21.3]	5.7 [1.3–15.1]	76 [53–98]
*p* value for interaction^d^		0.33	0.76	0.32	0.11
Body mass index					
≥ 25 kg/m^2^	2/14 (14.3)	344 [286–377]	15.4 [6.1–34.9]	7.3 [5.5–16.6]	73 [44–100]
< 25 kg/m^2^	12/46 (26.1)	310 [245–354]	7.0 [2.0–19.9]	4.3 [1.2–14.3]	79 [57–98]
*p* value for interaction^d^		0.40	0.78	0.97	0.46
Hypertension					
No	10/44 (22.7)	310 [264–361]	7.0 [3.1–23.3]	4.9 [1.4–15.6]	76 [52–100]
Yes	4/16 (25.0)	320 [213–369]	11.9 [3.2–22.9]	9.6 [2.06–13.3]	86 [63–98]
*p* value for interaction^d^		0.20	0.051	0.09	0.99
Diabetes					
No	12/50 (24.0)	311 [252–370]	9.6 [3.6–26.6]	6.5 [1.8–17.3]	77 [52–97]
Yes	2/10 (20.0)	320 [248–360]	6.4 [2.0–10.3]	2.8 [0.3–9.8]	86 [59–100]
*p* value for interaction^d^		0.07	0.82	0.89	0.16
Preoperative calcium channel blocker					
No	11/49 (22.4)	311 [266–367]	7.3 [2.2–22.4]	5.2 [1.4–15.3]	76 [51–100]
Yes	3/11 (27.3)	245 [211–330]	11.2 [6.7–25.7]	9.9 [4.7–11.9]	86 [65–97]
*p* value for interaction^d^		0.37	0.06	0.12	0.56
Type of surgery					
Hepatectomy	7/22 (31.8)	277 [247–354]	6.5 [2.1–17.0]	3.8 [1.6–7.3]	76 [48–89]
Colorectal resection	2/11 (18.2)	290 [203–328]	10.2 [2.0–25.9]	5.2 [0.7–11.9]	60 [26–100]
Gastrectomy	2/10 (20.0)	370 [284–380]	21.3 [3.3–30.5]	13.4 [2.5–22.8]	67 [63–76]
Esophagectomy	1/7 (14.3)	336 [331–354]	7.3 [4.6–17.2]	6.9 [3.1–16.6]	100 [95–100]
Pancreaticoduodenectomy	2/5 (40.0)	480 [343–486]	15.7 [12.6–22.3]	12.6 [2.7–19.4]	71 [45–90]
Lobectomy	0/4 (0.0)	180 [164–198]	6.1 [5.4–7.2]	5.1 [3.6–6.6]	96 [56–98]
Biliary tract surgery	0/1 (0.0)	314	49.0	44.7	91
*p* value for interaction^d^		0.82	0.15	0.73	0.26
Surgical technique					
Open	8/33 (24.2)	304 [245–343]	6.3 [2.1–18.4]	3.5 [1.4–11.0]	66 [51–100]
Laparoscopic-assisted	5/26 (19.2)	334 [268–373]	10.0 [5.3–33.8]	7.1 [2.2–22.8]	77 [58–97]
Robotic-assisted	1/1 (100.0)	335	12.6	12.6	100
*p* value for interaction^d^		0.58	0.94	0.55	0.51
Epidural analgesia					
No	7/31 (22.6)	325 [235–377]	9.7 [4.2–24.6]	7.3 [1.3–17.1]	78 [61–100]
Yes	7/29 (24.1)	309 [253–343]	6.7 [2.2–22.4]	4.0 [1.7–11.0]	63 [46–93]
*p* value for interaction^d^		0.41	0.65	0.17	0.19

MI, myocardial injury; AKI, acute kidney injury.

a Data were presented as frequency (percentage), or median [interquartile range].

b Hypotension with reduced cardiac output indicates that hypotension episodes (mean arterial pressure < 65 mmHg lasting for at least 1 min) were accompanied by reduced cardiac output (cardiac index <2.5 L/min/m^2^ or < 10% below preoperative baseline). The mean arterial pressure and cardiac index were simultaneously monitored and recorded.

c The percentage of hypotension with reduced cardiac output equals the duration of hypotension with reduced cardiac output / duration of total hypotension * 100% for each patient.

d The *p* values for interaction indicates the heterogenetic effects of different exposures (i.e., duration of anesthesia, duration of hypotension, duration of hypotension with reduced cardiac output, and percentage of hypotension with reduced cardiac output) on postoperative MI or AKI across subgroups, which were assessed by testing the significance of the exposure-by-group interaction in the multivariable logistic regression models.

### The burden of hypotension and its subtypes in patients with or without myocardial or acute kidney injury

Overall, 14 (23.3%) patients met the diagnostic criteria for myocardial injury or acute kidney injury after noncardiac surgery. Among them, 8 (13.3%) patients met the diagnostic criteria for postoperative myocardial injury within 3 days after surgery, while 6 (10.0%) patients met the KDIGO criteria for acute kidney injury (Table 1 and Supplementary Table S1).

The information regarding the burden of perioperative hypotension in patients with or without postoperative myocardial injury or acute kidney injury is presented in Table 2. Although the frequency of hypotension was comparable in patients with or without postoperative myocardial injury or acute kidney injury (median [interquartile range], 3 [1–9] vs. 3 [1–8]), the duration of hypotension might be longer in patients with postoperative myocardial injury or acute kidney injury (median [interquartile range], 13.6 [2.3–28.3] vs. 7.4 [3.6–22.4] minutes). The AUC of hypotension might be larger in patients with postoperative myocardial injury or acute kidney injury (median [interquartile range], 5,670 [789–11,494] vs. 3,699 [922–10,147] mmHg*min).

The duration and AUC of hypotension with reduced cardiac output might be larger in patients with postoperative myocardial injury or acute kidney injury (median [interquartile range]; duration: 8.6 [0.4–14.6] vs. 5.6 [1.8–14.3] minutes; AUC: 4,051 [70–7,842] vs. 2,999 [433–6,009] mmHg*min).

However, the percentages of hypotension with reduced cardiac output were similar in patients with or without postoperative myocardial injury or acute kidney injury (median [interquartile range], 77 [51–86] vs. 76 [54–100] %).

### Results of sensitivity analysis: the frequency and duration of hypotension based on different hypotension definitions

The sensitivity analyses were presented in Supplementary Tables S2, S3. When using the threshold of mean arterial pressure < 60 mmHg to define hypotension, 41 (68.3%) patients experienced perioperative hypotension. The median frequency of hypotension for each patient was 2 [interquartile range, 0–4], and the median duration for each patient was 4.2 [interquartile range, 0.0–9.5] minutes. Reduced cardiac output was present during 82% of the hypotension duration (Supplementary Table S2). Although no significant difference was found, the AUC of hypotension (i.e., AUC of mean arterial pressure below 60 mmHg) might be slightly larger in patients with postoperative myocardial injury or acute kidney injury compared with those without (median [interquartile range], 2,212 [0–4,351] vs. 1,505 [0–3,699] mmHg*min).

When using the threshold of mean arterial pressure < 55 mmHg, 32 (53.3%) patients experienced hypotension. The median frequency of hypotension for each patient was 1 [interquartile range, 0–2], and the median duration for each patient was 1.3 [interquartile range, 0.0–3.5] minutes. Reduced cardiac output was present during 98% of the hypotension duration (Supplementary Table S3). Patients with postoperative myocardial injury or acute kidney injury might show a larger AUC of hypotension (median [interquartile range], 369 [0–2001] vs. 108 [0–1,456] mmHg*min).

## Discussion

### Key results

This is the first prospective study investigating the subtypes of hypotension during the whole surgical period in patients undergoing moderate-to-high-risk noncardiac surgery. Among the 60 patients included in the analysis, 83.3% experienced perioperative hypotension with most of the hypotension periods being accompanied by cardiac output reduction. The hypotension and hypotension with cardiac output reduction most frequently occurred within 30 min after anesthesia induction. In addition, a higher burden of hypotension was observed in patients who suffer from postoperative myocardial injury or acute kidney injury, regardless of the hypotension subtypes. However, the percentage of different hypotension subtypes did not differ in patients with or without postoperative myocardial or acute kidney injury.

### Interpretation

The results from this study highlight the importance of decreased cardiac output in perioperative hypotension among patients who underwent moderate-to-high-risk noncardiac surgery. A series of elements may contribute to cardiac output reduction, including patient-related factors (e.g., preexisted cardiac disease); anesthesia-related factors (e.g., pharmacological sympathetic nerve-blocking effects of anesthetic and analgesic drugs, inadequate intravascular volume); and surgery-related factors (e.g., heart compression during lung or esophageal surgery, surgical bleeding) (1, 30). As most perioperative hypotension was observed to be accompanied by reduced cardiac output, this may serve as an important clue to medical caregivers that improving cardiac output might be critical when treating perioperative hypotension. Vasopressors and inotropes were used to treat hypotension in the acute care settings, but there was no definitive evidence for the choice of them during surgery. The scenario in the perioperative settings can be entirely different for those who are admitted to the intensive care unit (31). For critically ill patients, especially those with sepsis, systemic vasodilation (32) is the leading cause of hypotension, and norepinephrine is the recommended first-line treatment (33). Our results suggested the clinical implications that using vasopressors to increase vascular resistance should not be the only way to treat perioperative hypotension. An optimal choice of hypotension treatment should be investigated in future studies.

A significant hypotension burden is suggested among patients older than 45 years and undergoing moderate-to-high-risk noncardiac surgery. The incidence of hypotension (83.3%) in this study resonates with a large-sample retrospective study based on 22,109 patients from 11 hospitals in the United States (the incidence of hypotension ranges from 83.2 to 91.6%) (24). The duration of hypotension in our cohorts (median, 8 min) is lower than the previous report (median, 14), which may be due to the attention paid to hypotension and early intervention by anesthesiologists in recent years (1). The previous report was based on data obtained in 2017, while our cohort was enrolled in 2021 and 2022.

Hypotension was observed to occur most frequently within 30 min after anesthesia induction. This observation was consistent with a previous study which showed the unit duration of hypotension before incision was much longer than after incision (34). We also observed that post-induction hypotension often accompanied by reduced cardiac output. Saugel et al. found that anesthetic induction reduced systemic vascular resistance index but not cardiac index (35). Our results did not contradict this finding because the previous study measured the hemodynamic change, but we described the status of low cardiac output. Actually, our study showed consistent results with another study which observed a significant decrease in the cardiac index after anesthesia induction with propofol (36).

This study also systematically evaluated the incidence of postoperative myocardial injury and acute kidney injury, and preliminarily explored the relationship between hypotension subtypes and postoperative myocardial or acute kidney injury. The incidence of postoperative myocardial injury in our cohort was 13%, slightly lower than that reported in a previous study (18%) (37). The incidence of acute kidney injury in the present study is 10%, which is consistent with recent reports (38, 39). A previous study suggested that the association between intraoperative mean arterial pressure and postoperative complications is independent of cardiac index among patients who had undergone noncardiac surgery (25). It is similar to our observation whereby the incidence of hypotension with or without reduced cardiac output was similar in patients with postoperative complications.

### Generalizability

The reduction of postoperative organ injury and mortality after surgery is the ultimate goal of perioperative medicine (40), and an optimized perioperative hypotension management seems to be a promising way in achieving these goals (2). However, the criteria and strategies in treating hypotension are commonly prescribed to be subjective, which leads to a wide variation in clinical practice. Based on results described above, we planned to move forward to a larger cohort study to explore the association between hypotension subtypes and postoperative myocardial or acute kidney injury, which could provide evidence to improve perioperative blood pressure management.

### Limitations of the study

This study has its own limitations. Most importantly, discrepancy in the definitions of hypotension and reduced cardiac output could alter the results. However, currently there is no absolute definition for hypotension or reduced cardiac output. To minimize biases, the most accepted threshold for perioperative hypotension (1) and the most recently reported criteria for cardiac output reduction (25) were chosen. Secondly, this is a pilot study with a relatively small sample size. Although we observed a trend that patients with myocardial injury or acute kidney injury had more exposure to hypotension, the results could be underpowered. Considering the limited number of patients, we did not perform multivariable adjustment for confounding factors. Therefore, drawing a more definitive conclusion would necessitate further studies with a larger sample size. Thirdly, a noninvasive monitor (CNAP system) was deployed to obtain information on cardiac output, which might not be interchangeable with other invasive methods (41). Nevertheless, recent reviews have described the feasibility of noninvasive cardiac output monitoring systems (42, 43), and previous studies observed a good concordance rate and an acceptable agreement between continuous noninvasive cardiac output and transpulmonary thermodilution (44). Fourth, we included patients undergoing various types of noncardiac surgeries. Heterogeneity could exist in perioperative hemodynamic changes. Therefore, we did a subgroup analysis to show the hypotension distribution in different types of patients (Table 3). Fifth, we did not assess the troponin levels before surgery for every patient. Although we diagnosed postoperative myocardial injury in alignment with previous recommendations, it’s important to acknowledge that elevated troponin levels in certain patients before surgery could potentially impact the study results.

### Conclusion

Perioperative hypotension was often accompanied by cardiac output reduction in moderate-to-high-risk noncardiac surgical patients. Although patients with postoperative myocardial or acute kidney injury seem to experience more hypotension exposure in all subtypes, the relationship between hypotension subtypes and postoperative myocardial or acute kidney injury still needs further exploration due to the pilot nature of this study.

## Data availability statement

The raw data supporting the conclusions of this article will be made available by the authors, without undue reservation.

## Ethics statement

The studies involving humans were approved by this study was approved by the Institutional Review Board of the First Affiliated Hospital of Sun Yat-sen University (Chairperson: Churong Yan) before the recruitment of any subject (No. [2021]-764; approval date: November 2, 2021). The studies were conducted in accordance with the local legislation and institutional requirements. Written informed consent for participation in this study was provided by the participants' legal guardians/next of kin.

## Author contributions

XZ: Conceptualization, Data curation, Formal analysis, Funding acquisition, Investigation, Methodology, Software, Validation, Visualization, Writing – original draft, Writing – review & editing. YZ: Data curation, Investigation, Methodology, Writing – original draft, Writing – review & editing. MK: Data curation, Investigation, Methodology, Writing – original draft, Writing – review & editing. ZWa: Data curation, Resources, Writing – original draft, Writing – review & editing. QH: Data curation, Resources, Writing – original draft, Writing – review & editing. ZWe: Resources, Software, Writing – original draft, Writing – review & editing. JC: Data curation, Writing – original draft, Writing – review & editing. YS: Formal analysis, Writing – original draft, Writing – review & editing. SW: Resources, Supervision, Writing – original draft, Writing – review & editing. CH: Data curation, Funding acquisition, Methodology, Resources, Writing – original draft, Writing – review & editing. WH: Conceptualization, Methodology, Resources, Supervision, Writing – original draft, Writing – review & editing.
