# Supplementary material for: The exploration of perioperative hypotension subtypes: a prospective, single cohort, observational pilot study
Source: Front Med (Lausanne). 2024 Jun 17;11:1358067. doi: 10.3389/fmed.2024.1358067 (PMC11215119; doi:10.3389/fmed.2024.1358067)
Supplement: Supplementary file 1 [file Table_1.docx]

**Table S1.** Postoperative information

| Variable | Patients (n=60) ^a^ |
| --- | --- |
| **Incidence** |  |
| Postoperative myocardial injury or acute kidney injury, n (%) | 14 (23.3) |
| Myocardial injury within postoperative 3-day, n (%) | 8 (13.3) |
| Diagnosed on postoperative day 1 | 3 (5.0) |
| Diagnosed on postoperative day 2 | 1 (1.7) |
| Diagnosed on postoperative day 3 | 4 (6.7) |
| Acute kidney injury within postoperative 7-day, n (%) | 6 (10.0) |
| KDIGO stage 1 | 5 (8.3) |
| KDIGO stage 2 | 0 (0.0) |
| KDIGO stage 3 | 1 (1.7) |
| Diagnosed on postoperative day 1 | 4 (6.7) |
| Diagnosed on postoperative day 2 | 1 (1.7) |
| Diagnosed on postoperative day 3 | 1 (1.7) |
| Diagnosed after postoperative day 3 | 0 (0.0) |
| **Troponin T and creatinine** |  |
| Troponin T, ng/mL | |
| Postoperative 24-hour | 0.009 [0.005 – 0.013] |
| Postoperative 48-hour | 0.008 [0.005 – 0.013] |
| Postoperative 72-hour | 0.007 [0.005 – 0.014] |
| Highest within postoperative 72-hour | 0.011 [0.007 – 0.018] |
| Creatinine, umol/L | |
| Postoperative 24-hour | 65.0 [58.1 – 79.8] |
| Postoperative 48-hour | 62.8 [51.9 – 78.3] |
| Postoperative 72-hour | 58.4 [49.1 – 74.8] |
| Highest within postoperative 7-day | 73.2 [60.7 – 88.4] |

KDIGO, Kidney Disease: Improving Global Outcomes criteria.

^a^ Data were presented as frequency (percentage) or median [interquartile range].

**Table S2.** Sensitivity analysis: Frequency and duration of hypotension (defined as mean arterial pressure of **< 60mmHg** for more than 1 minute) ^a^

| Variable | Total (n=60) | With postoperative MI or AKI (n=14) | Without postoperative MI or AKI (n=46) | P value |
| --- | --- | --- | --- | --- |
| **Overall hypotension** | | | |  |
| Patients with hypotension, n (%) | 41 (68.3) | 9 (64.3) | 32 (69.6) | 0.71 |
| Frequency of hypotension ^b^, n | 2 [0 – 4] | 3 [0 – 5] | 1 [0 – 3] | 0.57 |
| Duration of hypotension, min | 4.2 [0.0 – 9.5] | 5.5 [0.0 – 10.1] | 3.8 [0.0 – 8.2] | 0.64 |
| Maximum duration of a single episode of hypotension, min | 2.7 ± 2.8 | 2.5 ± 2.4 | 2.8 ± 2.9 | 0.78 |
| AUC of hypotension ^c^, mmHg*min | 1678 [0 – 3807] | 2212 [0 – 4351] | 1505 [0 – 3699] | 0.78 |
| **Hypotension subtypes** | | | |  |
| Hypotension with reduced cardiac output ^d^ | | | |  |
| Duration, min | 3.0 [0.0 – 7.0] | 4.0 [0.0 – 6.7] | 2.6 [0.0 – 7.0] | 0.92 |
| Maximum duration of a single episode, min | 1.9 ± 2.3 | 2.0 ± 2.4 | 1.9 ± 2.3 | 0.94 |
| AUC ^e^, mmHg*min | 1107 [0 – 2553] | 1217 [0 –3663] | 1107 [0 –2438] | 0.92 |
| Hypotension without reduced cardiac output ^f^ | | | |  |
| Duration, min | 0.0 [0.0 – 3.0] | 0.0 [0.0 – 5.1] | 0.0 [0.0 – 1.8] | 0.61 |
| Maximum duration of a single episode, min | 0.8 ± 1.8 | 0.6 ± 1.0 | 0.9 ± 2.0 | 0.59 |
| AUC, mmHg*min | 0 [0 –1378] | 0 [0 – 1545] | 0 [0 – 868] | 0.74 |
| **Percentage of hypotension subtypes ^g^** (The duration of hypotension subtypes / duration of total hypotension * 100% for each patient) | | | | |
| Percentage of hypotension with reduced cardiac output, % | 82 [56 – 100] | 71 [50 – 100] | 87 [57 – 100] | 0.58 |
| Percentage of hypotension without reduced cardiac output, % | 18 [0 – 44] | 29 [0 – 50] | 13 [0 – 43] | 0.58 |

MI, myocardial injury; AKI, acute kidney injury; AUC, area under the curve; MAP, mean arterial pressure.

^a^ Data were presented as frequency (percentage), mean ± standard deviation, or median [interquartile range].

^b^ The frequency of hypotension indicated the total count of hypotension episodes for each patient.

^c^ AUC indicates the area under the curve below the MAP threshold (60mmHg), reflecting the products of hypotension duration and extent.

^d^ Hypotension with reduced cardiac output indicates that hypotension episodes (MAP <60mmHg lasting for at least one minute) were accompanied by reduced cardiac output (cardiac index <2.5 L/min/m^2^ or <10% below preoperative baseline). The MAP and cardiac index were simultaneously monitored and recorded.

^e^ The AUC of hypotension with reduced cardiac output indicates the AUC below the MAP threshold (55mmHg), while the low MAP was accompanied by a reduced cardiac output. It reflects the products of cardiac output reduction-related hypotension duration and extent.

^f^ Hypotension without reduced cardiac output indicates the hypotension episodes were not accompanied by reduced cardiac output (cardiac index ≥2.5 L/min/m^2^ or ≥90% of the preoperative baseline).

^g^ The percentage of hypotension with reduced cardiac output equals the duration of hypotension with reduced cardiac output / duration of total hypotension * 100% for each patient. However, we excluded the patients with a total hypotension duration <5 minutes when calculating the percentage, considering that short episodes might cause extreme percentage (0% or 100%) and biased the results.

**Table S3.** Sensitivity analysis: Frequency and duration of hypotension (defined as mean arterial pressure of **< 55mmHg** for more than 1 minute) ^a^

| Variable | Total (n=60) | With postoperative MI or AKI (n=14) | Without postoperative MI or AKI (n=46) | P value |
| --- | --- | --- | --- | --- |
| **Overall hypotension** | | | |  |
| Patients with hypotension, n (%) | 32 (53.3) | 8 (57.1) | 24 (52.2) | 0.74 |
| Frequency of hypotension ^b^, n | 1 [0 – 2] | 1 [0 – 2] | 1 [0 – 1] | 0.59 |
| Duration of hypotension, min | 1.3 [0.0 – 3.5] | 1.5 [0.0 – 4.0] | 1.3 [0.0 – 3.4] | 0.62 |
| Maximum duration of a single episode of hypotension, min | 1.3 ± 1.4 | 1.6 ± 1.6 | 1.3 ± 1.4 | 0.53 |
| AUC of hypotension ^c^, mmHg*min | 143 [0 – 1533] | 369 [0 –2001] | 108 [0 – 1456] | 0.57 |
| **Hypotension subtypes** | | | |  |
| Hypotension with reduced cardiac output ^d^ | | | |  |
| Duration, min | 0.0 [0.0 – 3.1] | 0.0 [0.0 – 3.2] | 0.0 [0.0 – 2.2] | 0.87 |
| Maximum duration of a single episode, min | 1.0 ± 1.4 | 1.2 ± 1.5 | 0.9 ± 1.3 | 0.55 |
| AUC ^e^, mmHg*min | 0 [0 – 1206] | 0 [0 – 2001] | 0 [0 – 1037] | 0.70 |
| Hypotension without reduced cardiac output ^f^ | | | |  |
| Duration, min | 0.0 [0.0 – 0.4] | 0.0 [0.0 – 0.0] | 0.0 [0.0 – 0.4] | 0.66 |
| Maximum duration of a single episode, min | 0.4 ± 0.9 | 0.4 ± 0.8 | 0.4 ± 0.9 | 0.87 |
| AUC, mmHg*min | 0 [0 –58] | 0 [0 –0] | 0 [0 – 115] | 0.63 |
| **Percentage of hypotension subtypes ^g^** (The duration of hypotension subtypes / duration of total hypotension * 100% for each patient) | | | | |
| Percentage of hypotension with reduced cardiac output, % | 98 [31 – 100] | 100 [24 – 100] | 92 [39 – 100] | 0.83 |
| Percentage of hypotension without reduced cardiac output, % | 2 [0 – 69] | 0 [0 – 76] | 8 [0 – 61] | 0.83 |

MI, myocardial injury; AKI, acute kidney injury; AUC, area under the curve; MAP, mean arterial pressure.

^a^ Data were presented as frequency (percentage), mean ± standard deviation, or median [interquartile range].

^b^ The frequency of hypotension indicated the total count of hypotension episodes for each patient.

^c^ AUC indicates the area under the curve below the MAP threshold (55mmHg), reflecting the products of hypotension duration and extent.

^d^ Hypotension with reduced cardiac output indicates that hypotension episodes (MAP <55mmHg lasting for at least one minute) were accompanied by reduced cardiac output (cardiac index <2.5 L/min/m^2^ or <10% below preoperative baseline). The MAP and cardiac index were simultaneously monitored and recorded.

^e^ The AUC of hypotension with reduced cardiac output indicates the AUC below the MAP threshold (55mmHg), while the low MAP was accompanied by a reduced cardiac output. It reflects the products of cardiac output reduction-related hypotension duration and extent.

^f^ Hypotension without reduced cardiac output indicates the hypotension episodes were not accompanied by reduced cardiac output (cardiac index ≥2.5 L/min/m^2^ or ≥90% of the preoperative baseline).

^g^ The percentage of hypotension with reduced cardiac output equals the duration of hypotension with reduced cardiac output / duration of total hypotension * 100% for each patient. However, we excluded the patients with a total hypotension duration <5 minutes when calculating the percentage, considering that short episodes might cause extreme percentage (0% or 100%) and biased the results.
