# Supplementary material for: The exploration of perioperative hypotension subtypes: a prospective, single cohort, observational pilot study
Source: Front Med (Lausanne). 2024 Jun 17;11:1358067. doi: 10.3389/fmed.2024.1358067 (PMC11215119; doi:10.3389/fmed.2024.1358067)
Supplement: Supplementary file 2 [file Table_2.pdf]

The Exploration of Perioperative Hypotension Subtypes (PHS):

A Prospective, Single Cohort, Observational Pilot Study

CLINICAL PROTOCOL
(Clinical Investigational Plan)
Effective Date: Oct/01/2021

Prepared by:

Wenqi Huang, MD
Xu Zhao, MD

Department of Anesthesiology
The First Affiliated Hospital, Sun Yat-sen University
Guangzhou, China

Table of Contents

1. Background ... 3
2. Study Objectives ... 4
3. Study Design ... 4
4. Study Method ... 5
    4.1 Subject enrollment, informed consent, and subject identification ... 5
    4.2 Perioperative monitoring ... 5
    4.3 Blood sampling and data collection ... 5
    4.4 Sample size calculation ... 6
    4.5 Statistical analysis plan ... 6
5. Bias, quality control, and risk ... 7
References ... 8

1. Background

Reducing postoperative complications and mortality is the eternal theme for improving perioperative medical quality.[1] Postoperative organ injury, especially myocardial injury and acute kidney injury, is the most important cause of perioperative complications and death.[2] In recent years, a large number of studies have shown that perioperative hypotension will increase the incidence of postoperative myocardial injury,[3-8] kidney injury[5,6,9,10] and stroke,[11-13] as well as the mortality rate.[2-4] Hemodynamic instability is an important characteristic of surgical patients, and varying degrees of hypotension are common during anesthesia and surgery. Hypotension can decrease organ perfusion pressure and then affect organ perfusion, leading to organ or tissue ischemia.[2] However, there were still some studies that suggested no association between hypotension and adverse outcomes.[14,15] Results from randomized controlled trials did not consistently prove that maintaining higher blood pressure improves postoperative outcomes.[16-18]

These contradictory findings can be attributed to many reasons, among which the heterogeneity of hypotension itself may be an important issue. Blood pressure is the result of orderly integrations of multiple hemodynamic elements. Cardiac output and systemic vascular resistance are the two leading determinants. However, capturing advanced hemodynamic parameters, such as cardiac output requires special devices. The complexity and costs of these devices limit their routine use in clinical practice. Therefore, although previous retrospective studies are based on large-sample cohorts, the information related to blood flow or organ perfusion is still lacking.

Researchers have recognized the heterogeneity of hypotension in recent years.[19] Hypotension does not always lead to organ hypoperfusion. It may not affect or may even increase organ perfusion, depending on the relative changes between the perfusion pressure and the regional vascular resistance and pressure autoregulation.[19] Therefore, hypotension may be classified as different subtypes based on the hemodynamic status, pathophysiological mechanisms, and impact on organ perfusion. However, studies investigating the frequency and distribution of hypotension subtypes were lacking, and the association between different hypotension subtypes and postoperative organ injuries is also unknown. Clarifying the characteristics of different types of hypotension can help us better understand the mechanism of hypotension and the main reasons causing hypotension-related organ injury after surgery. This study will investigate the frequency and distribution of different subtypes of hypotension in the perioperative setting, explore the association between different subtypes of hypotension and organ injuries, and finally, find more optimized strategies for perioperative blood pressure management.

2. Study Objectives

1. Investigate the frequency of hypotension during noncardiac surgery in our institute.
2. Describe the pattern and distribution of hypotension subtypes during noncardiac surgery.
3. Investigate the incidence of myocardial injury and acute kidney injury after noncardiac surgery in our institute.
4. Explore the association between different hypotension subtypes and organ injuries.

3. Study Design

This is a prospective, single cohort, observational pilot study. Patients undergoing noncardiac surgery at the First Affiliated Hospital, Sun Yat-sen University, will be enrolled in the study from October 2021 to February 2022.

The inclusion criteria are: 1) age ≥45 years; 2) moderate or high-risk noncardiac surgery (for example, gastrointestinal surgery, hepatobiliary surgery, urology surgery, esophagectomy, pneumonectomy, lung or liver transplantation); 3) general anesthesia; 4) surgery duration expected to last ≥2 hours from skin incision; 5) planned postoperative hospitalization ≥3 days; 6) written informed consent.

The exclusion criteria are: 1) participating in another interventional study; 2) receiving low-risk surgery only (for example, superficial surgery, breast surgery, thyroid surgery, minor plastic surgery, transurethral resection of the prostate); 3) confirmed to be pregnant and/or nursing mothers; 4) emergency surgery; 5) having severe comorbidities (clinically important intra-cardiac shunts, aortic stenosis with valve area ≤ 1.5 cm^2, moderate to severe aortic regurgitation, moderate to severe mitral regurgitation, moderate to severe mitral stenosis, persistent atrial fibrillation, acute congestive heart failure); 6) previously receiving heart valve surgery, coronary artery bypass grafting, percutaneous coronary intervention, pacemaker implantation, or ICD implantation; 7) patient in whom an intraoperative mean arterial pressure target will be <65 mmHg (including controlled hypotension); 8) with an intra-aortic balloon pump (IABP) or ventricular assist device(s); 9) requiring multiple vasoactive agents and known diagnosis of ongoing active sepsis.

4. Study Method

Before recruitment of any subjects into the study, written approval of the protocol and informed consent will be obtained from the Institutional Review Board (IRB).

4.1 Subject enrollment, informed consent, and subject identification

The primary investigator will review the subject's medical records for eligibility. Once the potential eligibility has been determined, the study will be discussed with the subject, and interest in study participation will be determined. Potential subjects will be fully informed of the study's purpose and nature, including potential risks and benefits. Subjects who voluntarily agree to participate in the trial will be asked to sign and date the informed consent form (ICF). The ICF will explain the study to the subject in lay terms and inform the subject that they may withdraw from the study at any time and for any reason. The potential study participant must sign the ICF before the subject undergoes the planned surgical intervention. A copy of the signed and dated ICF will be provided to the subject.

Each screened subject will be provided a sequential research ID. The research ID number will be recorded on all study documents and link the study documents to the subject's name and medical records. The subject's name should not be recorded on any study document other than the informed consent form to maintain confidentiality. There are no follow-up requirements for subjects who withdraw from the study before 24-hour after surgery. These subjects will also not be counted toward the eligible subjects enrolled in the trial.

4.2 Perioperative monitoring

All patients will receive routine monitoring, including electrocardiography, pulse oximetry, invasive/noninvasive blood pressure, and end-tidal carbon dioxide. In addition, patients will receive an electroencephalogram monitor (Narcotrend®; MonitorTechnik, Bad Bramstedt, Germany) and a hemodynamic monitor (CNAP®; CNSystems Medizintechnik GmbH, Graz, Austria). The finger cuff of the CNAP® monitor will be placed on the hand, while the cuff used for noninvasive blood pressure measurement was placed on the upper arm for calibration. If the patient receives an arterial line, the finger cuff will be placed on the side contralateral to the arterial line. A research laptop will be used to capture all real-time data. The monitoring and data recording will start before anesthesia induction and stop 10-15 minutes after extubation.

4.3 Blood sampling and data collection

Venous blood samples will be drawn from each patient at four time points: 1) postoperative 24-hour, 2) postoperative 48-hour, 3) postoperative 72-hour, and 4) postoperative 7-day or the last

day before the patient's hospital discharge. The concentration of serum troponin T, troponin I, and creatinine will be determined by a researcher who will not involve in the patient care.

Other data to be collected includes:

1. Preoperative data: 1) age, sex, height, weight, the American Society of Anesthesiologists (ASA) physical status classification; 2) comorbidity and previous history: cardiovascular system (hypertension, coronary heart disease, angina, myocardial infarction, arrhythmia, heart valve disease, congestive heart failure, peripheral vascular disease, cerebrovascular disease), non-cardiovascular system (chronic obstructive pulmonary disease, diabetes, hyperlipidemia, gastrointestinal disease, liver disease, kidney disease, malignant tumor), history of surgery, history of smoking; 3) medications: angiotensin-converting enzyme inhibitor, angiotensin receptor inhibitor, β -blockers, calcium channel blockers, diuretics, statins, aspirin; 4) preoperative laboratory results.
2. Intraoperative data: 1) monitoring data (including baseline before induction and intraoperative time-series): of inspired oxygen, end-tidal carbon dioxide; 2) medication: drugs for anesthesia induction and maintenance, vasoactive agents, etc.; 3) input and output: crystals and colloid, urine output, estimated blood pressure, heart rate, pulse oxygen saturation, cardiac output, stroke volume, systemic vascular resistance, respiratory rate, the fraction blood loss; 4) events: anesthesia induction time, pneumoperitoneum time, change of posture time, clamping time.

4.4 Sample size calculation

This is the first study exploring the frequency and distribution of different hypotension subtypes during surgery, and there is no reference for the rates of hypotension subtypes. Therefore, this study should be regarded as a pilot study. According to current guidelines for sample size estimation for pilot studies[20] and the surgical volume of our hospital, we plan to enroll 60 patients.

4.5 Statistical analysis plan

The hypotension will be classified into different subtypes depends on whether the cardiac output or systemic vascular resistance decreases.

1. Describe the distribution of perioperative hypotension subtypes during noncardiac surgery by presenting and visualizing the frequency and tendency;
2. Describe the hypotension and hypotension subtypes stratified based on different surgical types, surgical stages, and populations;
3. Describe the incidence of myocardial injury and acute kidney injury after noncardiac surgery in our institute;
4. Explore the association between different hypotension subtypes (types and durations) and postoperative troponin level, creatinine level, rate of myocardial injury, and rate of acute kidney injury, based on linear regression or logistic regression. Multivariable analysis and propensity-score matching will be used to control for confounders.

5. Bias, quality control, and risk

The data collection process will be done independently by two researchers to ensure data accuracy. A quality control project (dealing with missing and abnormal data) will be performed prior to the analysis of perioperative monitoring data. A research training will be conducted for all research staffs before data acquisition to ensure consistency.

References

1. Nepogodiev D, Martin J, Biccard B, Makupe A, Bhangu A. Global burden of postoperative death. Lancet 2019;393:401.
2. Sessler DI, Khanna AK. Perioperative myocardial injury and the contribution of hypotension. Intensive Care Med 2018;44:811-22.
3. Abbott TE, Pearse RM, Archbold RA, et al. A prospective international multicentre cohort study of intraoperative heart rate and systolic blood pressure and myocardial injury after noncardiac surgery: results of the VISION study. Anesthesia and analgesia 2018;126:1936.
4. Sessler DI, Meyhoff CS, Zimmerman NM, et al. Period-dependent associations between hypotension during and for four days after noncardiac surgery and a composite of myocardial infarction and death: a substudy of the POISE-2 trial. Anesthesiology 2018;128:317-27.
5. Salmasi V, Maheshwari K, Yang D, et al. Relationship between intraoperative hypotension, defined by either reduction from baseline or absolute thresholds, and acute kidney and myocardial injury after noncardiac surgery: a retrospective cohort analysis. Anesthesiology 2017;126:47-65.
6. Walsh M, Devereaux PJ, Garg AX, et al. Relationship between intraoperative mean arterial pressure and clinical outcomes after noncardiac surgery: toward an empirical definition of hypotension. Anesthesiology 2013;119:507-15.
7. Van Waes JA, Van Klei WA, Wijeysundera DN, Van Wolfswinkel L, Lindsay TF, Beattie WS. Association between intraoperative hypotension and myocardial injury after vascular surgery. Anesthesiology 2016;124:35-44.
8. Hallqvist L, Mårtensson J, Granath F, Sahlén A, Bell M. Intraoperative hypotension is associated with myocardial damage in noncardiac surgery: an observational study. European Journal of Anaesthesiology| EJA 2016;33:450-6.
9. Sun LY, Wijeysundera DN, Tait GA, Beattie WS. Association of intraoperative hypotension with acute kidney injury after elective noncardiac surgery. Anesthesiology 2015;123:515-23.
10. Hallqvist L, Granath F, Huldt E, Bell M. Intraoperative hypotension is associated with acute kidney injury in noncardiac surgery. European journal of anaesthesiology 2018;35:273-9.
11. Group PS. Effects of extended-release metoprolol succinate in patients undergoing noncardiac surgery (POISE trial): a randomised controlled trial. The Lancet 2008;371:1839-47.
12. Sabaté S, Mases A, Guilera N, et al. Incidence and predictors of major perioperative adverse cardiac and cerebrovascular events in non-cardiac surgery. Br J Anaesth 2011;107:879-90.
13. Bijker JB, Persoon S, Peelen LM, et al. Intraoperative hypotension and perioperative ischemic stroke after general surgery: a nested case-control study. The Journal of the American Society of Anesthesiologists 2012;116:658-64.
14. Hsieh JK, Dalton JE, Yang D, Farag ES, Sessler DI, Kurz AM. The association between mild intraoperative hypotension and stroke in general surgery patients. Anesthesia & Analgesia 2016;123:933-9.

15. Babazade R, Yilmaz HO, Zimmerman NM, et al. association between intraoperative low blood pressure and development of surgical site infection after colorectal surgery. Annals of surgery 2016;264:1058-64.
16. Carrick MM, Morrison CA, Tapia NM, et al. Intraoperative hypotensive resuscitation for patients undergoing laparotomy or thoracotomy for trauma: early termination of a randomized prospective clinical trial. Journal of Trauma and Acute Care Surgery 2016;80:886-96.
17. Futier E, Lefrant J-Y, Guinot P-G, et al. Effect of individualized vs standard blood pressure management strategies on postoperative organ dysfunction among high-risk patients undergoing major surgery: a randomized clinical trial. Jama 2017;318:1346-57.
18. Vedel AG, Holmgaard F, Rasmussen LS, et al. High-target versus low-target blood pressure management during cardiopulmonary bypass to prevent cerebral injury in cardiac surgery patients: a randomized controlled trial. Circulation 2018;137:1770-80.
