# Supplementary figures and images for: The exploration of perioperative hypotension subtypes: a prospective, single cohort, observational pilot study
Source: Front Med (Lausanne). 2024 Jun 17;11:1358067. doi: 10.3389/fmed.2024.1358067 (PMC11215119; doi:10.3389/fmed.2024.1358067)

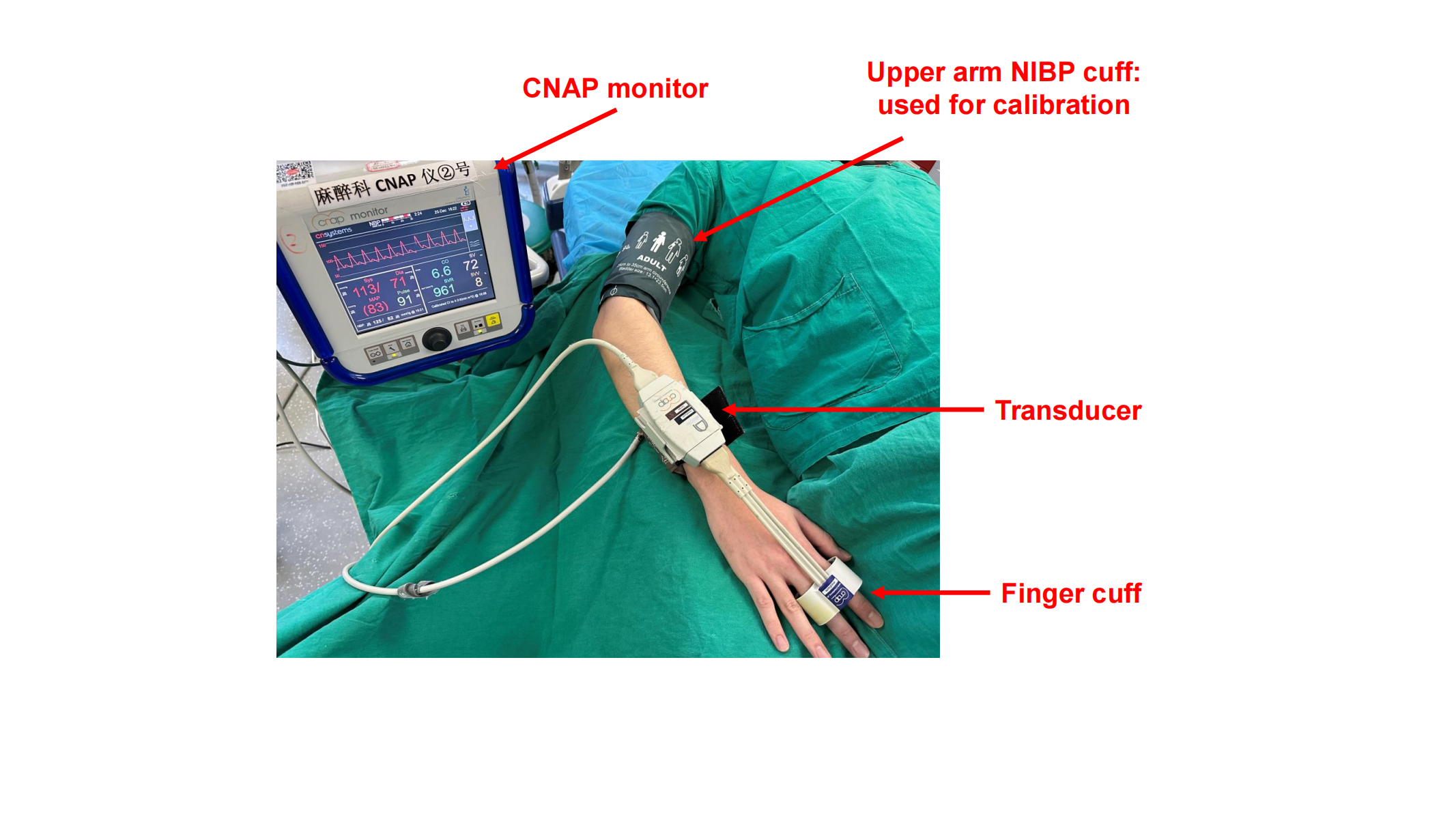

Supplement: Supplementary file 3 [file Image_1.tif]
